# Neighborhood perceptions and hypertension among low-income black women: a qualitative study

**DOI:** 10.1186/s12889-016-3741-2

**Published:** 2016-10-12

**Authors:** Maliyhah Al-Bayan, Nadia Islam, Shawneaqua Edwards, Dustin T. Duncan

**Affiliations:** 1 Meharry Medical College, Nashville, TN USA; 2 Department of Population Health, Spatial Epidemiology Lab, New York University School of Medicine, 227 East 30th Street, 6th Floor, Room 621, New York, NY 10016 USA

**Keywords:** Blood pressure, Hypertension, Neighborhoods, Low-income housing, Qualitative research, Black women

## Abstract

**Background:**

The majority of studies examining the role of neighborhoods and hypertension-related outcomes have been quantitative in nature and very few studies have examined specific disadvantaged populations, including low-income housing residents. The objective of this study was to use qualitative interviews to explore low-income Black women’s perceptions of their neighborhoods and to understand how those perceptions may affect their health, especially as it relates to blood pressure.

**Methods:**

Seventeen Black female participants, living in public housing communities in New York City, completed one semi-structured, audiotaped interview in July of 2014. All interviews were transcribed, coded, and analyzed for emerging themes using N’Vivo 10 software.

**Results:**

Three major themes emerged: (1) social connectedness, (2) stress factors, and (3) availability of food options. For example, factors that caused stress varied throughout the study population. Sources of stress included family members, employment, and uncleanliness within the neighborhood. Many participants attributed their stress to personal issues, such as lack of employment and relationships. In addition, the general consensus among many participants was that there should be a greater density of healthy food options in their neighborhoods. Some believed that the pricing of fresh foods in the neighborhoods should better reflect the financial status of the residents in the community.

**Conclusions:**

Various neighborhood influences, including neighborhood disorder and lack of healthy food options, are factors that appear to increase Black women’s risk of developing high blood pressure. Implications of this research include the need to develop interventions that promote good neighborhood infrastructure (e.g. healthy food stores to encourage good nutrition habits and well-lit walking paths to encourage daily exercise), in addition to interventions that increase hypertension awareness in low-income neighborhoods.

## Background

Approximately 70 million American adults, or one in every three, are living with hypertension, [1] a leading risk factor for heart disease and cardiovascular disease. Additionally, one in three American adults are living with prehypertension, [1] a condition in which blood pressure readings are higher than normal, but not high enough to be classified as hypertension. Both hypertension and prehypertension are particularly common in Black adults, who more often develop elevated blood pressure at an earlier age than Hispanic and White adults [1]. The prevalence of hypertension in Black adults in the United States is among the highest in the world, and is particularly high among Black women [2]. Although many studies have analyzed hypertension in the Black population, most have focused on Black individuals as a whole [3], as opposed to studying hypertension by gender in this vulnerable population subgroup.

A significant body of evidence also indicates that hypertension disproportionately affects low-income populations [4]. One study found that low-income housing residents in Boston reported substantially poorer health than did other city residents across a variety of chronic conditions, including hypertension [5]. The prevalence of self-reported hypertension among low-income populations was more than two times higher than the rate of hypertension among non-low-income housing residents [5]. Low-income housing residents also reported higher levels of physical inactivity [5], placing them at risk for developing elevated blood pressure. Furthermore, previous research has shown an association between low socioeconomic status and reduced function of the kidneys [6], which help to regulate blood pressure within the body.

Determinants of hypertension have predominantly focused on individual-level risk factors, such as race/ethnicity and gender. Little research has examined how the neighborhood context can influence hypertension. However, emerging research demonstrates that neighborhood-level factors, such as walking environment and availability of healthy foods in neighborhoods, can affect hypertension [7]. These neighborhood factors may influence hypertension through their effects on physical activity and diet. Indeed previous research demonstrates that residents living in neighborhoods of low walkability often fail to meet physical activity recommendations [8], which negatively impacts blood pressure over time [9] and thus increases the risk of developing hypertension. Well-advertised fresh food markets and fewer fast food restaurants in neighborhoods promote healthy eating amongst residents [10], thus reducing the risk of developing hypertension. Poor neighborhood safety and social cohesion may affect hypertension through physical activity and/or psychosocial stress [7].

While informative, the majority of studies examining the role of neighborhoods and hypertension-related outcomes have been quantitative in nature and very few studies have examined specific disadvantaged populations, including low-income housing residents. Furthermore, few studies have qualitatively investigated the mechanisms through which neighborhoods influence hypertension and related health outcomes among such disadvantaged populations. Qualitative approaches may be particularly useful in identifying relationships between socioeconomic and neighborhood factors and their effect on hypertension in low-income communities. Generally, qualitative studies are highly effective in identifying intangible factors, whose role in research topics cannot be easily measured or quantified and helpful in hypothesis generation for topics less frequently studied. They can also help us to interpret and better understand the complex reality of a given situation and thus can yield important implications for quantitative data [11–13].

Using a qualitative approach, the purpose of this study was to examine the perceptions of neighborhood influences on health among Black female low-income housing residents’ with and without hypertension. By analyzing accounts of hypertension versus normal blood pressure, we sought to identify pathways of risk and further the understanding of how Black female residents’ of low-incoming housing perceive the relationship between neighborhood factors and hypertension. The use of a qualitative study was expected to be highly effective in obtaining specific information about the values, opinions, behaviors, and social contexts of this understudied population.

## Methods

### Study design

This analysis was part of a larger quantitative study – the NYC Low-income Housing, Neighborhoods and Health Study (*n* = 120), which was designed to understand connections between neighborhood characteristics and cardiovascular disease risk among low-income individuals in New York City, using geospatial technologies [14, 15]. Recruitment for the parent quantitative study was conducted through various advertising and marketing methods, which included distributing flyers outside of three low-income housing developments in three different Manhattan neighborhoods and one low-income housing development in Queens. Flyers were posted in community locations (e.g. local stores) and verbal communication with community members aided in recruitment to neighbors and friends. Participants were considered eligible for participation in the parent study if they reported living in low-income housing (e.g. public housing) in New York City, were 18 years of age or older, could speak English, self-reported not being pregnant, self-reported no restrictions to usual physical activity, and were willing to wear a Global Positioning Systems (GPS) device (on their person; e.g. in their pocket) for a week. Participants were given a small incentive for completing baseline surveys and health screenings. They were given a larger incentive for returning the GPS device and completing the post-GPS survey. Participants were invited to participate in the qualitative study after they completed a weeklong GPS protocol. In addition to the eligibility criteria previously articulated, participants for the qualitative study had to be Black and female. Sixty-three participants from the parent study were eligible to participate in the qualitative study. We limited the qualitative study to seventeen participants, who were selected at random from the group of eligible participants by creating a random sample in an Excel spreadsheet. We yielded a participation rate of 27 %. All of the women who were asked to participate in the qualitative study agreed (100 % agreement rate). The small sample size was primarily due to restraints of time, budgeting, the use of a single interviewer, and sample saturation. Sample saturation occurs when there is a lack of new concepts emerging from the interviews [16, 17]. The 17 participants each received a $10 Starbucks gift card for taking part in the qualitative interviews. Each participant was assigned a study number to protect her anonymity. Written informed consent was obtained from all participants prior to collection of data. The Human Subjects Protection committee at New York University School of Medicine reviewed and approved the protocols for this research.

### Interview guide and data collection

To explore participants’ perceptions of neighborhoods in relation to hypertensive risk and behavior, we developed a semi-structured interview guide. Each participant completed one semi-structured [18], audiotaped interview in July of 2014, lasting approximately 30 min each. The interview guide questions are shown in Table 1. The interviews included open-ended questions regarding various neighborhood factors such as perceived neighborhood safety, perceived neighborhood disorder, as well as health behaviors including engagement in physical activity and levels of daily stress—many of which have been identified in previous research as risk factors for hypertension. Interviews were audio recorded with an Olympus VN-722PC voice recorder. The lead researcher (MA-B) conducted the interviews at community locations and at our research office. She is not from the study area and did not know the participants prior to the interview. However, she is a Black woman, like the participants, which likely fostered greater rapport, trust, and openness in communication [19].

**Table 1:** Interview questions

1. How long have you lived in this neighborhood?
2. Can you describe some of the major changes you would like to see in the neighborhood?
3. How safe do you feel in your current neighborhood?
4. How much time do you spend outside in your neighborhood? What are some activities you like to do while outside?
5. Can you describe the food options that are available to you and others in your neighborhood?
6. Can you describe your attitudes towards fast food?
7. Can you describe the recreational options that are available to you and others in your neighborhood?
8. Can you briefly describe what you know about hypertension, also known as high blood pressure?
9. Can you overview for me your typical daily routine from the time you wake up to the time you go to bed?
10. How would you describe what is most important to you in a day?
11. What factors do you consider when purchasing food?
12. Can you describe factors in your life that cause you the most stress?

### Blood pressure

Using standard protocols, trained research assistants measured the participant’s blood pressure. Blood pressure was measured with a Welch Allyn Vital Signs 300 monitor [20–25]. Blood pressure is summarized by two measurements, systolic blood pressure and diastolic blood pressure, both measured in millimeter of mercury (mmHg). Systolic blood pressure refers to arterial pressure during the contraction of the heart, while diastolic pressure refers to arterial pressure between beats when the heart fills with oxygenated blood from the lungs and deoxygenated blood from rest of the body. Hypertension is a medical condition in which the force of blood against artery walls is elevated, and is classified as a systolic blood pressure greater than 140 mmHg, or a diastolic blood pressure greater than 90 mmHg. For our purposes, pre-hypertension is classified as a systolic blood pressure between 120–139 mmHg, or a diastolic blood pressure between 80–89 mmHg. Normal blood pressure is classified as a systolic blood pressure less than 120 mmHg and a diastolic blood pressure less than 80 mmHg [26].

### Data analyses

Interviews were transcribed verbatim and coded. Codes were identified based on data collected during the interviews and were grouped under three major themes, which provided the framework for analyzing responses among Black women living in low-income housing. N’Vivo 10 software was used to analyze the qualitative data gathered in this study—which is consistent with previous qualitative research [27, 28]. Analyses of the data from transcriptions were performed by a single coder, through direct content analysis using a constant comparative [29] and narrative analysis approach [30, 31]. The “constant comparison” approach is a method of explanation building in which the findings of an initial case are compared to a provisional category, revised as necessary and then other details or new cases are then compared against the revision and revised again as needed [32]. Central to this process is the “thematic” coding scheme. Specifically, we developed an initial set of codes, informed by the in-depth interview guides. The goal of this analysis was the systematic identification of themes and the specification of relationships among these themes and/or with contextual factors [33, 34]. To ensure reliability and consistency of the coding process, intra-coder procedures were established, in which the same researcher coded the data at different points in time.

## Results

### Participant information

Our study included 17 low-income Black female participants (see Table 2). Sixteen of the participants were 21 to 65 years old. The remaining participant was identified as an outlier due to her age of 81 years. The mean age for participants was 47. Systolic blood pressures ranged from 106 to 163 mmHg, yielding a mean of 135 mmHg. Diastolic blood pressures ranged from 55 to 101 mmHg, yielding a mean of 85 mm Hg. In the sample, 41 % of participants were normotensive, while 24 % were pre-hypertensive and 35 % were hypertensive. Family history of hypertension was self-reported in nine (53 %) of the seventeen interviews.

**Table 2:** Participant information

Participant #	Age	Blood pressure status
1	45	Hypertension
2	40	Normal
3	81	Hypertension
4	53	Hypertension
5	65	Hypertension
6	25	Normal
7	57	Normal
8	34	Normal
9	24	Normal
10	49	Pre-hypertension
11	54	Hypertension
12	43	Pre-hypertension
13	49	Pre-hypertension
14	21	Normal
15	36	Hypertension
16	54	Pre-hypertension
17	53	Hypertension

### Qualitative findings

Three major themes emerged: (1) social connectedness, (2) stress factors, and (3) individual food preferences.

### Theme 1: Social connectedness

Social connectedness describes the degree to which participants communicated with and existed within (e.g. physically and socially) their low-income housing communities, and the areas immediately surrounding their housing communities. It was gauged by participants’ community involvement, desire for community change, and knowledge of current neighborhood activities. Participants were asked about the amount of time they have lived in their current low-income housing communities, the average amount of time they spent outside in their neighborhoods each day, outdoor activities that they enjoyed to do in their neighborhoods, and some of the changes that they would like to see in their neighborhoods.

There was variability in the length of time participants lived in low-income housing. Generally, hypertensive participants had lived in low-income housing for a longer period of time and spent more time outside in their neighborhood each week when compared to their non-hypertensive counterparts. They were also very involved in their housing communities, and actively sought improvements to their respective communities.

*“I’m in charge of the outside of Queensbridge, so what I do is I go from building to building trying to get people to sit tenant patrol, try to involve them in the neighborhood, see what particular issues are, see if I can be a liaison between the housing and the residents, and just try to empower the people in the community.” (Participant 17; hypertensive)*

*“Well I usually walk. I talk. I take care of my grandson so I have him outside or whatever I do with my community. If, you know, if they have anything that needs to be done I get in involved with the community.” (Participant 11; hypertensive)*

Many participants passionately described changes they would like to see in their housing communities. They expressed genuine concern for the quality of their neighborhoods and the conditions of their housing communities.

*“The major change that I want to see in my neighborhood is more things for the youth. You know, have their time occupied so they are not out there trying to do different things to get themselves in trouble. Anything to occupy their time, so they're not worried about going out there robbing and stealing or doing you know things that are unnecessary. So anything to help the youth I’m with. I think more things for them is the best thing for the neighborhood.” (Participant 15; hypertensive)*

*“Uhh the biggest one is that I’d like for New York City Housing Authority to remove garbage bins that they have coming at the entrance of each building. That garbage bins. I don’t like too much dog poop out there. Tenants are supposed to clean up behind themselves but they don’t.” (Participant 3; hypertensive)*

Other participants were largely less connected to their neighborhood and social resources. Many spent most of their time in other neighborhoods or inside of their apartments, as illustrated in the following quote:

*“I just go to the store and back for real…I don’t say nothing to nobody.” (Participant 8; normotensive)*

Participants were also asked to describe the recreational options available in their neighborhoods. It was of interest to us to find that participants with high blood pressures were generally more aware of the options available in their neighborhoods, such as parks and community centers.

“*There’s a park. They got…Central Park so they got bicycle riding there. Um, what else? They got the museum. I’m not too far from the Museum of Natural History. They got this chocolate factory right there on the corner of my block… I just found out there’s a pool not too far off from where I live.…Um, but my neighborhood, there’s a lot of things. I just gotta find them, you know.” (Participant 4; hypertensive)*

Many of the younger, normotensive participants were also aware of parks that were present in their neighborhoods but generally were not aware of specific recreation programs. One participant, in particular, expressed her concern regarding the lack of recreational options available for the youth in the community. Another reported that she would be more inclined to take advantage of certain recreational options if they were more affordable.

*“I would like to see in my neighborhood a little more exercise uh activities for everyone, not just where you have to pay and make it feasible that we can all afford to get a little healthy, you know, in my neighborhood. Um, it’s sort of rich in it’s own way. It’s rich in tradition but there are new people moving in and prices are changing, and in order to stay healthy, some of us cannot afford that. If you’re putting up a gym, it would be nice that you know maybe two days out of a week it would be free for the people in the neighborhood and if you could prove that you’re in the neighborhood, just you know, things like that would be excellent.” (Participant 7; normotensive)*

*“I can say it’s not a lot of things for the kids to do so that’s why they get in so much trouble…I would put more community activities for the teenagers to get off the street and stuff like that. Maybe some teenager activities because that’s where the police are attracted to the most for some reason and I just feel like if there was more stuff for the teenagers to do it’ll be better.” (Participant 9; normotensive)*

Overall, participants with higher blood pressures seemed to spend more time outside in their neighborhood and actively sought to empower the community. They were also very involved in their communities. Non-hypertensive participants tended to be less involved in the community, spent time in other neighborhoods, and more time in their homes, and were more likely to avoid social interactions within their community. Consistently, hypertensive participants expressed genuine concern for the quality of their neighborhoods and the conditions of their housing communities. They passionately called for greater opportunities for the youth. They expressed a vast depth of knowledge regarding local museums, parks, and youth recreational activities as well. Of note, these differences were simply observations and may not be related to the hypertensive status of our participants.

### Theme #2: Stress factors

Stress was a common theme that emerged in many interviews. However, the factors that caused stress varied throughout the study population. Many participants commonly attributed most of their stress to undesirable neighborhood conditions, such as uncleanliness.

*“Uh only if I’m going into the lobby or coming down or coming in and you have that weed smell. That…just upsets me. It just sends a (pause) gives me a headache. I thank God that nobody on my floor smokes weed because I’ve been in it already. I’ve been right there because I don’t tolerate that foolishness. But the lobby, you do come in and sometimes you can get, what is it, a contact high or whatever. You smoking and that bothers me, particularly if I’m going to church and I’m dressed. I don’t want to smell like weed.” (Participant 3; hypertensive)*

*“Too dirty. Clean up…the streets. It’s not that bad in Staten Island. It’s just too junky. Like I think some places in front of buildings and…in certain areas in front of stores should be an um…they should have to like power wash their sidewalk. It’s just dirty, like.” (Participant 1; hypertensive)*

*“I don't like it over there…For me, I don't care that it’s the project that we're living in. Don't put your garbage in front of the dumpster. You put it in the dumpster dodo brain. … Don't throw your garbage in front of the building throw it in the trash bin. … We walked to the supermarket at like 2 [in the morning]…and all these rats. You should have seen them.” (Participant 10; pre-hypertensive)*

Some of our study participants, especially those who were considered very active members in their housing communities, were frustrated by the lack of effort and support they receive from their neighbors. One participant spoke about the efforts she put forth to make improvements in her community and to empower the other residents. Her frustration stemmed from their complacency and resistance to, what she considers, positive change. Other participants found the community’s youth particularly stressful due to their lack of guidance and perceived lack of motivation to improve their quality of living.

*“Um I would say trying to empower people in my community is very difficult these days. People are so stuck in their ways, in their actions that they’re not willing to just think about change. The older folks feel like they’re older and it doesn’t matter too much…The younger folks feel like it’s just a waste of time because the results are not gonna come…People want instant gratification. People have a sense of entitlement, so all those things are very frustrating for me when you are trying to empower people in your community.”* *(Participant 17; hypertensive)*

*“When I see our young black guys out on the street. Young black women out on the street with no direction, um with no structure that kind of um bothers me. That kind of hits home because when I see them I think of my of my family of my children, so I try to do my best as a mom and live a positive life so that I can have positive kids.” (Participant 12, pre-hypertensive)*

Some participants attributed their stress to more personal issues, such as problems occurring within their families and lack of employment.

*“Um not having a job at the moment. That’s the most stressful thing because it’s like I want money now so. Uh that’s it, not having a job. That’s the most stressful thing, cause once I get a job then I…I think everything will be a little better.” (Participant 9; normotensive)*

*“Honestly, my grandmother stresses me out. I don’t know if she is a factor but she stresses me out all of the way…She just nags.” (Participant 14; normotensive)*

*“My husband and this damn surgery again [are stressful]. Um, he had surgery on his back two months ago…so I don’t know how it is going to go…and he is angry with everybody in the world because he says he should have been healed by now.” (Participant 10; pre-hypertensive)*

Study participants did not express traditional neighborhood factors as a source of stress. Instead, they expressed a sense of safety in their neighborhoods. Some participants reported high levels of perceived neighborhood safety and comfort in their housing communities, as illustrated in the following quotes:

*“Oh yes. I feel very safe. They don’t play with me.” (Participant 9; normotensive)*

*“I feel very safe. I feel very safe. I don’t walk in fear. I leave all my trust in God. You know what I’m saying? So my neighborhood, any neighborhood. Where is it actually 100 % safe? So that's how it is.” (Participant 11; hypertensive)*

All of the participants felt safe and comfortable in their neighborhoods.

### Theme #3: Availability of food options

Individual food choices can have important impacts on overall health. The general consensus among many participants was that there should be a greater density of healthy food options in their neighborhoods. Not only should the availability of healthy food options be increased, but they should also be more affordable to increase community members’ desire to buy healthier fresh foods over fast food. Participants acknowledged some of their food choices as being poor and detrimental to their health. Many expressed the desire to improve their eating habits and food choices, but only a few were taking action towards such measures.

Participants agreed that there were both fast food and fresh food options (e.g. supermarkets) in their communities. While some participants seemed to be more content with the availability of food options in their neighborhoods, many wanted to see differences in the quality and prices of the fresh food sold in their neighborhoods.

*“I live in a neighborhood with all the rich, White people and the prices – I can’t shop there. I can’t. I got to go all the way either to Harlem or to the Bronx. I can’t, I can’t…it’s too much. It’s too expensive. A pound of bacon ten dollars, and that’s it cause I live by D’Agostino’s and Gristedes and, you know, stuff like that. I can’t afford them. I’m on food stamps. I’m on welfare.” (Participant 4; hypertensive)*

*“Honey, it’s just so many [food options]. No, it’s just an overabundance of food options. I like to um, the seafood store on, um, 116th where you can actually get your raw things and they can steam it for you. I love those. Those are good.” (Participant 2; normotensive)*

One participant believed that she would have access to fresher food options if she lived in a different, higher-income community. She felt as though the residents of her community would benefit from more superior food options if they came together and voiced their concerns.

*“You know, you feel like because it’s a low-income community that they bring the worst of everything there. So you actually have to go outside of your community to get things that you feel are nutritious for your body…I know this for a fact because I go in different neighborhoods to shop so I’m able to see the variations between where I live … and other neighborhoods that I shop in…It just seems like they just give us the worst of just everything, and we accept it because if we got out there and we petitioned and we fought for a better quality of food, then I think there the change would come.” (Participant 17; hypertensive)*

Although we acknowledge that the differences between participants’ responses may not be related to their hypertensive status, we would like to highlight the fact that hypertensive participants, especially those that were previously aware of their high blood pressure status and those who were able to give accurate information about hypertension when asked during the interview, were more conscious of their food choices. They expressed mixed attitudes towards fast food and did not indulge in it as frequently, although it was readily available in their neighborhoods. They preferred to cook their own meals at home. Most non-hypertensive participants expressed content with their available food options and chose to indulge in them regularly. Participant 5 cooked mostly at home and was more conscious of her diet due to co-morbidities. Although participant 15 was also hypertensive, she valued convenience when choosing her meals. Her preference for convenience usually led her to eat fast foods that were not healthy for her.

*“I buy groceries a lot. I cook…I eat fast food but I usually eat things that are pretty healthy, you know, but I – I try to watch what I eat because I have the high blood pressure and the cholesterol.” (Participant 5; hypertensive)*

*“[I eat] fast food more because it’s more convenient. Once I get off work I don’t want to be in the house cooking. I’ll just go pick something up [and] go in the house. … It’s convenient; very fast but I know that it’s not healthy for me. I know I should be cooking and eating a home-cooked meal but it’s just convenient. That’s it. That’s the reason why I’ll get that before I’ll go in the house and cook.”* (*Participant 15; hypertensive*)

Many participants mentioned that they were aware that fast food options in their neighborhoods were not healthy. However, those that ate it often said it was a matter of convenience more than anything else.

## Discussion

In this study, we used qualitative interviews to identify Black female low-income housing residents’ perceptions of their neighborhoods and understand how neighborhood influences might affect their blood pressure. Three themes emerged from the interviews conducted: (1) social connectedness, (2) stress factors, and (3) availability of food options. A socio-ecological framework was applied to thematic analysis to indicate the impact of contextual factors. The first level of the framework identified individual level factors (i.e., stress). Interpersonal factors included social connectedness and support, forming the next level of the framework. The third level of our socio-ecological model identified environmental factors, such as neighborhood stress, one’s access to foods in her neighborhood and the types of foods that are available.

Social connectedness is a term used to describe the role participants played in their communities and the capacity to which they were involved in their neighborhoods. It describes the extent to which people in a community interact with one another, individually and in groups. Previous research has shown that social connectedness can have a direct impact on an individual’s health by affecting various factors that contribute to chronic disease prevention [35]. In our study, participants who have lived in their low-income housing communities for longer periods of time and had higher levels of connectedness with their neighborhoods tended to have a stronger sense of involvement with the situations that went on in their communities, and were more vocal about the problems within their neighborhoods that required improvement. Those that were found to have lower levels of social connectedness were generally more withdrawn from their neighborhoods, stayed inside of their apartments when not at work, and went to other neighborhoods for social activities. Previous studies have found that higher levels of perceived social connectedness are associated with lower blood pressure rates, better immune responses, and lower levels of stress hormones. On the contrary, lower levels of perceived social connectedness have been associated with obesity, high blood pressure, cancer, and diabetes [35]. Our findings suggest that an association may exist between increased social connectedness and higher blood pressures of Black women living in low-income housing. However, this requires future research attention.

Additionally, the third theme that emerged involved availability of food options. Most participants, who regularly ate fast food, acknowledged their awareness of its negative impact on their health; however, some participants noted that convenience and taste are both factors that lead them to eat fast food instead of home-cooked foods, which is consistent with prior research [36]. Some participants also believed that they were offered food of inferior quality when compared to the residents of higher income neighborhoods. It is possible that the poor quality of some of the available fresh foods may deter residents from indulging in them, causing them to become more inclined to pursue less healthy, processed food options. In addition, the women in our study were of lower socio-economic status. The higher cost of healthier food items may also lead some residents to sacrifice the quality of purchased groceries in order to maximize their budgets [37]. Future studies should seek to further explore the relationships between perceived availability of high-quality fresh foods in one’s neighborhood and blood pressure. Methods of improving the quality of food that is available in low-income neighborhoods without increasing costs should also be investigated.

Throughout the course of our study, notable differences were revealed between responses from hypertensive and non-hypertensive women. These differences have not been explored in current literature, to our knowledge. In our study, participants who have lived in their low-income housing communities for longer periods of time and had higher levels of connectedness with their neighborhoods tended to have higher blood pressure measurements. They also had a stronger sense of involvement with the situations that went on in their communities, and were more vocal about the problems within their neighborhoods that required improvement. Those with lower blood pressures were generally more withdrawn from their neighborhoods, stayed inside of their apartments when not at work, and went to other neighborhoods for social activities. In addition, hypertensive participants generally identified neighborhood conditions and the behaviors of residents in their housing communities as primary sources of stress, while non-hypertensive participants attributed most of their stress to their personal life (i.e. lack of employment). Hypertensive participants were very concerned about and impacted by the actions of their neighbors, troubled youth in the community, and overall conditions in the neighborhood. We also found that hypertensive participants who spent more time outside in their communities were more aware of the recreational options. It may be necessary to direct more efforts towards advertising these recreational options. Moreover, educating residents on the importance of daily physical activity and lowering the costs of such recreational services may aid in the maintenance of healthy blood pressure levels amongst low-income housing residents.

When considering the availability of food options in the neighborhood, hypertensive participants were more dissatisfied with the quality and pricing of food options in their neighborhoods and sought healthier options, especially those who were previously aware of their hypertensive blood pressure status and who were more knowledgeable about the risk factors of high blood pressure. Younger, non-hypertensive participants showed greater indulgence in the fast food options that were available in their neighborhoods. Hypertensive participants generally displayed higher, but still inadequate, levels of knowledge regarding hypertension [38] and high blood pressure when asked about it in their interviews. Their slightly higher level of hypertension awareness, when compared to their non-hypertensive counterparts, may have led to their preferred indulgence in and preference for healthier food choices within their neighborhoods. We suggest educating members of the observed at-risk populations in order to decrease the likelihood of high blood pressure development and to encourage positive health behaviors [39].

It is particularly difficult to generalize our findings to larger populations, but our study does enhance the literature on low-income Black women and social contextual factors in high blood pressure. Our investigation of Black women in low-income communities opens the gateway to explore various other health outcomes within this population. In addition, our study suggests possible research avenues for observational studies, including longitudinal studies, to explore the development of high blood pressure in Black women of low-income populations. Our research further implicates that perceived neighborhood stress related to community involvement and increased hypertension among Black women is a topic that needs to be explored in future research studies.

### Limitations

Limitations of the study include small sample size and self-reported information collected during the interviews. Due to the small sample size our research findings cannot be fully generalizable towards larger populations. However, this study does explore neighborhood perception, which can assist in the development of future studies that examine health outcomes in Black women in low-income communities. Multiple studies that explore these factors may aid in generalizing results towards larger populations. In addition, because this was a community-based study, we only measured blood pressure one time. Additional objectively-measured blood pressure values may provide a more accurate assessment of participants’ actual blood pressure, although having an objectively-measured blood pressure value compared to one’s self-report of blood pressure is a strength of this study [40]. Some factors of interviewer bias were controlled including our utilization of a Black female interviewer that was not from the study area. Participants’ general knowledge regarding the parent study may have led them to answer some of the health-related questions with responses that they deemed favorable. Self-selection of participants for this qualitative study was another limitation of the study.

## Conclusion

In this qualitative study, we found that various neighborhood influences, including neighborhood disorder and lack of healthy food options, are factors that appear to increase Black women’s risk of developing high blood pressure. Implications of this research include the need to develop interventions that promote good neighborhood infrastructure (e.g. healthy food stores to encourage good nutrition habits and well-lit walking paths to encourage daily exercise), which can in turn influences Black women’s health. Interventions should also aim to increase hypertension awareness in low-income neighborhoods.

## References

[1] Nwankwo T, Yoon SS, Burt V, Gu Q. Hypertension among adults in the United States: National Health and Nutrition Examination Survey, 2011–2012. NCHS Data Brief. 2013(133):1–8. 24171916

[2] Mozaffarian D, Benjamin EJ, Go AS, Arnett DK, Blaha MJ, Cushman M, de Ferranti S, Despres JP, Fullerton HJ, Howard VJ (2015). Heart disease and stroke statistics-2015 update: a report from the american heart association. Circulation.

[3] Centers for Disease Control and Prevention a (2010). A Closer Look at African American Men and High Blood Pressure Control: A Review of Psychosocial Factors and Systems-Level Interventions.

[4] Bowen DJ, Battaglia TA, Murrell SS, Bhosrekar SG, Caron SE, Smith E, Thomas G, Rorie JA, Maetschke LM, Goodman R (2013). What do public housing residents say about their health?. Prog Community Health Partnersh.

[5] Digenis-Bury EC, Brooks DR, Chen L, Ostrem M, Horsburgh CR (2008). Use of a population-based survey to describe the health of Boston public housing residents. Am J Public Health.

[6] Tamrat R, Peralta CA, Tajuddin SM, Evans MK, Zonderman AB, Crews DC (2015). Apolipoprotein L1, income and early kidney damage. BMC Nephrol.

[7] Mujahid MS, Diez Roux AV, Morenoff JD, Raghunathan TE, Cooper RS, Ni H, Shea S (2008). Neighborhood characteristics and hypertension. Epidemiology.

[8] Duncan DT, Méline J, Kestens Y, Day K, Elbel B, Trasande L, Chaix B (2016). Walk score, transportation mode choice, and walking among French adults: a GPS, accelerometer, and mobility survey study. Int J Environ Res Public Health.

[9] Li F, Harmer P, Cardinal BJ, Vongjaturapat N (2009). Built environment and changes in blood pressure in middle aged and older adults. Prev Med.

[10] Polsky JY, Moineddin R, Dunn JR, Glazier RH, Booth GL (2016). Absolute and relative densities of fast-food versus other restaurants in relation to weight status: Does restaurant mix matter?. Prev Med.

[11] Firestone WA (1987). Meaning in method: The rhetoric of quantitative and qualitative research. Educ Res.

[12] Gholizadeh M, Delgoshaei B, Gorji HA, Torani S, Janati A (2015). Challenges in Patient Discharge Planning in the Health System of Iran: A Qualitative Study. Global J Health Sci.

[13] Patton DU, Hong JS, Patel S, Kral MJ. A systematic review of research strategies used in qualitative studies on school bullying and victimization. Trauma Violence Abuse. 2015:1524838015588502. [Epub ahead of print] 10.1177/1524838015588502 26092753

[14] Duncan DT, Regan SD, Shelley D, Day K, Ruff RR, Al-Bayan M, Elbel B (2014). Application of global positioning system methods for the study of obesity and hypertension risk among low-income housing residents in New York City: a spatial feasibility study. Geospat Health.

[15] Duncan DT, Regan SD. Mapping multi-day GPS data: a cartographic study in NYC. J Maps. 2016;12(4):668–70. 10.1080/17445647.2015.1060180 PMC4896478 27293471

[16] Francis JJ, Johnston M, Robertson C, Glidewell L, Entwistle V, Eccles MP, Grimshaw JM (2010). What is an adequate sample size? Operationalising data saturation for theory-based interview studies. Psychol Health.

[17] O‘Reilly M, Parker N. ‘Unsatisfactory Saturation’: a critical exploration of the notion of saturated sample sizes in qualitative research. Qual Res. 2013;13(2):190–97.

[18] Franklin MM, Harden JK, Peters RM. Getting to Normal: Women‘s Experiences self-managing Their Perceived Blood Pressure Changes. J Cardiovasc Nurs. 2016;31(2):151–57. 10.1097/JCN.0000000000000223 25513987

[19] Kumanyika SK, Whitt-Glover MC, Gary TL, Prewitt TE, Odoms-Young AM, Banks-Wallace J, Beech BM, Halbert CH, Karanja N, Lancaster KJ (2007). Expanding the obesity research paradigm to reach African American communities. Prev Chronic Dis.

[20] Hess PL, Reingold JS, Jones J, Fellman MA, Knowles P, Ravenell JE, Kim S, Raju J, Ruger E, Clark S (2007). Barbershops as hypertension detection, referral, and follow-up centers for black men. Hypertension.

[21] Ravenell J, Thompson H, Cole H, Plumhoff J, Cobb G, Afolabi L, Boutin-Foster C, Wells M, Scott M, Ogedegbe G (2013). A novel community-based study to address disparities in hypertension and colorectal cancer: a study protocol for a randomized control trial. Trials.

[22] Victor RG, Ravenell JE, Freeman A, Leonard D, Bhat DG, Shafiq M, Knowles P, Storm JS, Adhikari E, Bibbins-Domingo K (2011). Effectiveness of a barber-based intervention for improving hypertension control in black men: the BARBER-1 study: a cluster randomized trial. Arch Intern Med.

[23] Alpert BS (2007). Validation of the Welch Allyn Spot Vital Signs blood pressure device according to the ANSI/AAMI SP10: 2002. Accuracy and cost-efficiency successfully combined. Blood Press Monit.

[24] Amoore JN (2012). Oscillometric sphygmomanometers: a critical appraisal of current technology. Blood Press Monit.

[25] Jones C, Taylor K, Poston L, Shennan A (2001). Validation of the Welch Allyn‘Vital Signs’ oscillometric blood pressure monitor. J Hum Hypertens.

[26] Krupp MA, and Milton John. Chatton: Systemic Hypertension. Lange Medical Pub 1974, Current Medical Diagnosis. New York City: The McGraw-Hill Companies.

[27] Sprague S, Swinton M, Madden K, Swaleh R, Goslings JC, Petrisor B, Bhandari M (2013). Barriers to and facilitators for screening women for intimate partner violence in surgical fracture clinics: a qualitative descriptive approach. BMC Musculoskelet Disord.

[28] Touboul P, Valbousquet J, Pourrat-Vanoni I, Alquier MF, Benchimol D, Pradier C (2011). [Adapting the environment to encourage the elderly to walk: a qualitative study]. Sante Publique.

[29] Hsieh H-F, Shannon SE (2005). Three approaches to qualitative content analysis. Qual Health Res.

[30] Mishler EG. Research interviewing: Context and narrative. Cambridge: Harvard University Press; 1991.

[31] Riessman CK. Narrative analysis, vol. 30. Newbury Park: Sage; 1993.

[32] Blumer H. Symbolic [nteractionism NJ. Englewood Cliffs: Prentice-Hall; 1969.

[33] Polkinghorne DE. Phenomenological research methods. In: Existential-phenomenological perspectives in psychology. Boston: Springer; 1989: 41–60.

[34] Atkinson R. The Life Story Interview. Thousand Oaks: Sage; 1988. p. 44.

[35] Uchino BN, Cacioppo JT, Kiecolt-Glaser JK (1996). The relationship between social support and physiological processes: a review with emphasis on underlying mechanisms and implications for health. Psychol Bull.

[36] Lucan SC, Barg FK, Karasz A, Palmer CS, Long JA (2012). Perceived influences on diet among urban, low-income African Americans. Am J Health Behav.

[37] Hernández D. Affording Housing at the Expense of Health: Exploring the Housing and Neighborhood Strategies of Poor Families. J Fam Issues. 2016;37(7):921–46. 10.1177/0192513X14530970 PMC4819250 27057078

[38] Teixeira Jde F, Goulart MR, Busnello FM, Pellanda LC (2016). Hypertensives‘ Knowledge About High-Sodium Foods and Their Behavior. Arq Bras Cardiol.

[39] McNaughton CD, Jacobson TA, Kripalani S (2014). Low literacy is associated with uncontrolled blood pressure in primary care patients with hypertension and heart disease. Patient Educ Couns.

[40] Williams JH, Duncan DT, Cantor J, Elbel B, Ogedegbe G, Ravenell J. A Comparison of Self-Reported and Measured Blood Pressure Status Among Low-Income Housing Residents in New York City. Journal of Health Disparities Research and Practice. In press.

